# Clinical Features in Patients with Long-Lasting Macrophagic Myofasciitis

**DOI:** 10.3389/fneur.2014.00230

**Published:** 2014-11-28

**Authors:** Muriel Rigolet, Jessie Aouizerate, Maryline Couette, Nilusha Ragunathan-Thangarajah, Mehdi Aoun-Sebaiti, Romain Kroum Gherardi, Josette Cadusseau, François Jérôme Authier

**Affiliations:** ^1^Faculty of Medicine, INSERM U955-Team 10, Créteil, France; ^2^Reference Center for Neuromuscular Diseases Garches-Necker-Mondor-Hendaye, Créteil, France; ^3^Neurology Department, Henri Mondor University Hospital, Créteil, France; ^4^Paris Est-Créteil University, Créteil, France

**Keywords:** aluminum, vaccines, myofasciitis, myalgias, chronic fatigue syndrome, mild cognitive impairment, neglected diseases, CCL2

## Abstract

Macrophagic myofasciitis (MMF) is an emerging condition characterized by specific muscle lesions assessing abnormal long-term persistence of aluminum hydroxide within macrophages at the site of previous immunization. Affected patients usually are middle-aged adults, mainly presenting with diffuse arthromyalgias, chronic fatigue, and marked cognitive deficits, not related to pain, fatigue, or depression. Clinical features usually correspond to that observed in chronic fatigue syndrome/myalgic encephalomyelitis. Representative features of MMF-associated cognitive dysfunction include dysexecutive syndrome, visual memory impairment, and left ear extinction at dichotic listening test. Most patients fulfill criteria for non-amnestic/dysexecutive mild cognitive impairment, even if some cognitive deficits appear unusually severe. Cognitive dysfunction seems stable over time despite marked fluctuations. Evoked potentials may show abnormalities in keeping with central nervous system involvement, with a neurophysiological pattern suggestive of demyelination. Brain perfusion SPECT shows a pattern of diffuse cortical and subcortical abnormalities, with hypoperfusions correlating with cognitive deficiencies. The combination of musculoskeletal pain, chronic fatigue, and cognitive disturbance generates chronic disability with possible social exclusion. Classical therapeutic approaches are usually unsatisfactory making patient care difficult.

Macrophagic myofasciitis (MMF) is an emerging condition, first reported in 1998 in adult patients presenting with chronic fatigue and arthromyalgias and defined by the presence of stereotyped inflammatory lesions at muscle biopsy (1). MMF lesion is very specific and characterized by (i) a focal epi-, peri-, and endomysial inflammatory infiltrate, mainly formed of large cohesive basophilic macrophages, with PAS-positive cytoplasmic content; (2) the presence of T-cells; and (3) the absence of significant myofiber injury (1). Afterwards, it was shown that MMF lesions correspond to long-lasting aluminic granulomas, resulting from previous intramuscular (i.m.) injection of aluminum-adjuvanted vaccines (2–4). Most recent works delineated the cognitive dysfunction associated with MMF (5, 6) and emphasized the neurological component of MMF-associated clinical syndrome (MACS) (4, 7). MMF belongs to rare diseases (Orpha number #ORPHA592, ICD-10 #M60.8, http://www.orpha.net/) and its prevalence is not exactly known. From 1993 to 2013, more than 600 cases were diagnosed in Henri Mondor hospital, and, to date, 293 patients are registered in our database. The place of muscle biopsy in the diagnosis approach of chronic myalgias has been regarded problematic and controversial (8), so explaining the delay elapsed between first symptoms and diagnosis in MMF patients (Table 1). The retrospective evaluation of 130 consecutive arthro-myalgic patients, previously immunized with aluminum-containing vaccine, showed that one-third had biopsy-proven MMF (7). Considering chronic musculoskeletal pain is very common in primary care practice, MMF appears still under-diagnosed in France and probably dramatically under-recognized in other countries where biopsy is not performed in the deltoid muscle (7).

**Table 1:** **Epidemiological data from 293 MMF patients registered in Henri Mondor hospital database**.

Age (years) Mean; median	52.7; 53.5
Sex ratio (M/F)	88/205
Mean number of aluminum-containing vaccines as indicated in vaccination booklets	5; range: 1–12 (data available in 183/293)
Mean persistence time of aluminum (months)	70
Mean delay between onset of symptoms and biopsy (months)	66.7
Symptoms	
Myalgias	254/278 (91%)
Fatigue	248/280 (89%)
Cognitive complaint	107/133 (80%)
Results of neuropsychological testing	*N* = 76
Cortico-subcortical profile	64/76 (84.2%)
Isolated callosal deconnexion	4/76 (5.3%)
Isolated dysexecutive syndrome	3/76 (3.9%)
Normal	5/76 (6.6%)
Abnormal evoked potentials	7/22 (31.8%)
Myopathic EMG	15/43 (34.9%)
Elevated CK serum levels	12/48 (25%)

## Meaning of Histological MMF Lesions

### Aluminum hydroxide is the causal factor of MMF lesions

At the beginning, the origin of MMF was unknown but an environmental cause, infectious, or toxic, seemed likely (1). Works conducted between 1998 and 2001 in Créteil and Bordeaux identified aluminum hydroxide-adsorbed vaccines as the causal factor lesions MMF (2). The electron microscopy scanning of biopsy samples from 40 consecutive patients showed the constant presence of spiculated inclusions within macrophages corresponding to aluminum hydroxide crystals. Aluminum hydroxide is a component used as immunostimulant adjuvant of many vaccines. MMF lesions were found only in the deltoid muscles (in adults) and quadriceps (in children), two conventional vaccinal sites, supporting the hypothesis of a local accumulation of vaccine-derived aluminum. The evaluation of the first 50 MMF patients established that all received at least one i.m. (i.m.) injection of an aluminic vaccine [hepatitis B virus (HBV): 84%; tetanus toxin (TT): 58%; hepatitis A virus (HAV): 19%] before the biopsy (time 3–96 months, median 36 months). Finally, in experimental conditions, the i.m. injection of aluminum hydroxide-adsorbed vaccine in rat induces typical MMF lesions at 21 and 28 days post-injection. Together, these results established that histopathological MMF lesions resulted from i.m. injection of aluminum hydroxide-adjuvanted vaccines (vaccines anti-tetanus, anti-hepatitis A, and anti-hepatitis B) and demonstrated the unexpected persistence over several years of this immunostimulant at site of previous injection (2, 9). Other i.m. injected products containing aluminum hydroxide may cause MMF, such as allergen preparations used for desensitization (personal observation).

### Pathological significance of MMF lesions

#### Persistence time of aluminum hydroxide after i.m. injection

**Figure 1:**
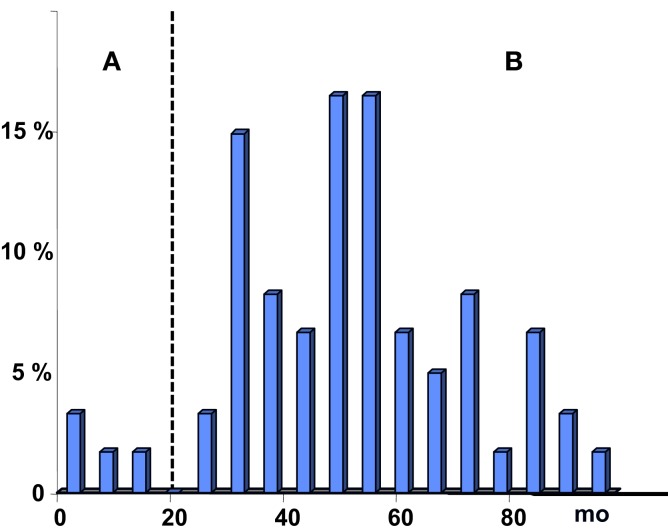
**Histogram showing the distribution of patients, according to the delay elapsed from last injection of aluminum hydroxide-containing vaccine to muscle biopsy evidencing MMF lesions**. For patients with a delay below 18 months (group A), it is not possible to consider MMF lesion as certainly pathological. In patients from group B, the delay is above 18 months indicating an abnormally protracted persistence time of aluminic granuloma.

The i.m. injection of a dose of a vaccine adsorbed on aluminum hydroxide is sufficient to induce MMF lesions in rats (3), macaque (10), and mouse (11). The biopersistence of such a lesion is much longer than originally thought. In rats, these lesions persist 1 year (3) and elimination kinetics depends on the genetic background. In macaques, 50% of injected animals had detectable lesions at 1 year. In human beings, the persistence time of post-vaccinal physiological tissue damage is not known. As long as we do not have non-invasive and reliable imaging technique to visualize MMF lesions with certainty, and that can substitute for biopsy, this data will be impossible to determine, and can only be based on projections, derived from experimental data obtained in animals. In 1999, the WHO asked for the mechanisms involved in the persistence of MMF lesion. It proposed that “A plausible possibility is the existence of a predisposed subset of individuals with impaired ability to clear aluminum from the deltoid muscle. Whether this reflects a macrophagic dysfunction of either genetic or acquired origin, or the tail end of a normal distribution describing the kinetics of aluminum clearance and the local tissue response to it in the general population, has not yet been defined” (9). In the current state of knowledge, it can be assumed that the presence of lesions MMF has no formal pathological significance if the biopsy was performed within one year after vaccination (provided that the biopsied muscle is the actual site of vaccine injection). If the delay is superior to 2 years, the presence of MMF lesions can be considered pathological. If one considers that the detection of MMF lesion is purely fortuitous phenomenon, the probability to find this lesion would be higher as the date of biopsy would be close to the date of vaccination. Examination of the distribution histograms of vaccination-biopsy delays goes against this hypothesis (Figure 1). The median delay elapsed between last vaccination and biopsy was found at 53 months (12) and the distribution profile shows a peak between 4 and 5 years after vaccination, in complete opposition to the model of “the tail of the normal distribution” (9). It must be noted that, in the small subset of patients, the delay was <1 year. In these cases, MMF lesion can not be considered pathological.

#### Case of other aluminum-based adjuvants

Aluminum salts other than aluminum hydroxide are used as vaccine adjuvants. For example, quadrivalent anti-human papilloma virus (HPV) vaccine available in France contains aluminum hydroxyphosphate. Several observations of girls developing fatigue/myalgias syndrome after HPV vaccination raised the question of possible MMF induced by this vaccine. At electron microscopy, the structure of aluminum hydroxyphosphate differs from that of aluminum hydroxide (13) suggesting different physicochemical properties. Macrophages in lesion were fluorescent with Morin technique, indicating the presence of aluminum. Time persistence of granuloma after quadrivalent HPV vaccine i.m. injection has not yet been determined in mouse to our knowledge. Moreover, further studies are needed to evaluate whether HPV vaccine may actually induce full-blown MMF in human beings.

#### MMF lesion, a post-vaccinal “tattoo”?

The evidence of an abnormally prolonged persistence (several years) of aluminum hydroxide in muscle tissue after i.m. injection, has led some authors to speak of “vaccination tattoo” (14, 15), considering MMF lesion as an inert vaccine scar. However, data from literature contradict this view and it seems somewhat unwise to regard protracted persistence of aluminum hydroxide within body as trivial. First, the majority of aluminum-containing vaccine receivers *do not* have long-standing MMF at muscle biopsy (7), considering MMF and non-MMF patients similar for age, sex-ratio, the number of aluminum-containing vaccines, and the delay between last vaccine injection and biopsy. Second, histopathological investigation indicates that MMF lesion is immunologically active (2), a finding consistent with the immunostimulant properties of aluminum hydroxide (16). Third, although it is true that MMF histological lesions have been observed only at usual sites of vaccinations so far, experiments performed in rodents (rabbits, mice) showed that the i.m. injection of aluminum hydroxide is accompanied by the diffusion of aluminum in the entire body, including a fraction of the aluminum that penetrates and persists in brain (11, 17).

## MMF-Associated Clinical Syndrome

Main clinical manifestations observed in adult patients with persistent MMF lesions at muscle biopsy are the following: (i) chronic *musculoskeletal pain* (arthromyalgias); (ii) chronic *fatigue*; and (iii) *cognitive disorders*. These manifestations were described in a series of studies published between 1998 and 2013 and also in two reports from French government agencies InVS and ANSM (formerly AFSSAPS) (18, 19). In contrast to what has been written (15), the analysis of these data allows defining quite precisely the clinical manifestations associated with MMF. MMF is classified as rare disease #ORPHA 592 in the Orphanet database (http://www.orpha.net/), which is the portal for information on rare diseases and orphan drugs, set up by INSERM (the French National Institute of Health and Medical Research), the French Directorate General for Health, and the European Commission. In the International Statistical Classification of Diseases and Related Health Problems (ICD), MMF is referenced as M608.

### Clinical manifestations in patients with MMF

As shown by retrospective analysis of 293 MMMF patients registered in our database (Table 1), patients are adults (mean age: 52.7 years), mainly women (sex ration M/F: 3/7). Mean delay between onset of symptoms and biopsy (diagnosis of MMF) was 66.7 months.

#### Musculoskeletal pain

The analysis of data from the literature (Table 2) and Henri Mondor database (Table 1) allows defining most representative features of MACS. The development of symptoms is usually slow, over several months. Diffuse myalgias were observed with a prevalence ranging from 55 to 96%. Myalgias have been recognized as cardinal symptoms since first description. The InVS study (18) showed that myalgias most often began distally in lower limbs, with subsequent progressive extension to the whole body. At physical examination, patients usually exhibit only few tender point sites, if not none. The tenderness at specific tender point sites is a characteristic feature of fibromyalgia, which are included in the clinical criteria for fibromyalgia proposed in 1990 by the American College of Rheumatology (ACR) (20). Logically, most MMF patients did not meet the 1990 ACR criteria for fibromyalgia (7). In a minority of patients, myalgias are the only functional complaints. Arthralgias are less frequently reported (14–84% depending on the series) and may sometime represent the only complaint. Spinal pain, especially dorsal, is also frequently observed. Finally, MMF patients typically present with diffuse arthromyalgias affecting both proximal and distal parts of lower and upper limbs and spine, pain being usually present at waking up and exacerbated by exercise and daily activity, leading to marked disability. At physical examination, muscle strength is usually normal, and the presence of a true deficit must prompt a search for a diffuse muscular inflammatory/dysimmune process (e.g., inclusions body myositis or autoimmune necrotizing myopathy) that may associates with MMF.

**Table 2:** **Prevalence of main clinical manifestations in MMF patients in published series**.

Reference	No. of patients	Myalgias (%)	Arthralgias (%)	Cognitive disturbances (%)	Psychiatric manifestations	Other
(1)	14	86	64	–	–	Weakness 43%
						Fever 28%
						Dyspnea 21%
(21)	12	92	58	–	–	Weakness 42%
						Fever 17%
						Spinal pain 17%
(22)	7	86	14	14	14	Multiple sclerosis (MS) 100%
(2)	50	94	–	–	–	Autoimmune diseases 34% (MS 12%)
(23, 24)	10	60	30	20	–	
(25)	30	87	57	50	50	Chronic fatigue syndrome 53%
(12)	30	88	57	50	53	Autoimmune diseases 19%
(26)	9	55	–	–	–	Neurological signs 44%
(5)	25	96	84	68 (Patients statement)	52	Cognitive dysfunction 100%
(27)	16	56	12.5	–	–	Chronic fatigue syndrome 50%
(18)	53	81	57	–	–	Headache 28%
						Dyspnea 27%
(19)	28	81	50	–	–	

#### Chronic fatigue

Chronic fatigue is the second cardinal symptom, with a prevalence ranging from 36 to 100% depending on the study (91% in InVS study). In a study conducted in 30 MMF patients (25), we found chronic (duration >6 months) fatigue in 28/30 patients (93%). Fatigue was considered severe and debilitating in 26/30 patients (87%), caused a significant reduction of activities in 24/30 (80%), was present more than half the time in 19/30 patients (63%), affected both physical and mental functioning in 16/30 patients (53%), and was not alleviated by rest in 13/30 patients (43%). The majority of patients (16/30, 53%) met the 1991 Oxford or 1994 CDC international criteria for chronic fatigue syndrome (25). In some patients, chronic fatigue may be the only symptom at onset and it may precede pain by several months.

#### MMF-associated cognitive dysfunction

**Figure 2:**
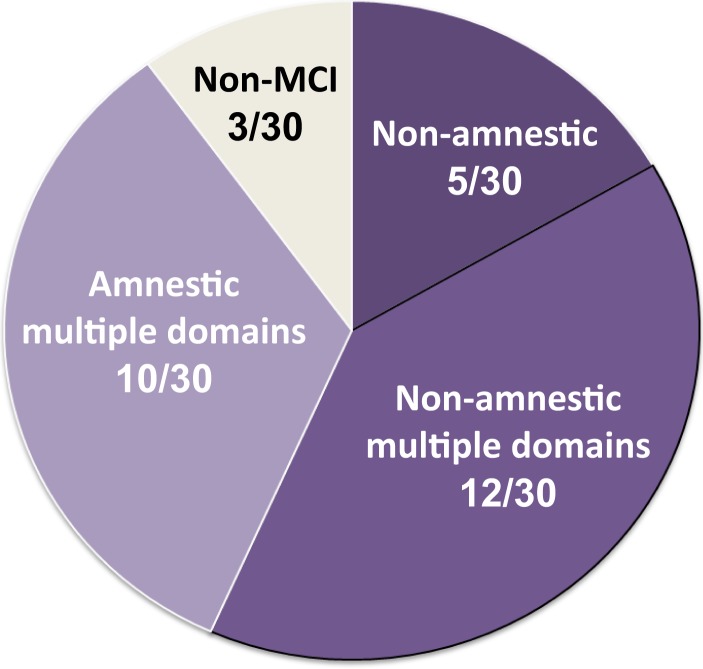
**Mild cognitive impairment (MCI) in MMF**. Classification of 30 MMF patients according to the neuropsychological profile of cognitive dysfunction. 27/30 (90%) fulfilled criteria for MCI, of amnestic type/multiple domains in 10/30 (33%) and of non-amnestic type in 17/30 (56.7%), multiple domains in 12/30 (40%). Results from Passeri et al. (6).

The third cardinal clinical manifestation in MMF is cognitive impairment (5, 6). This item has been neglected for a long time and was incorrectly considered as non-specific. Indeed, chronic pain, chronic fatigue states, and depressive syndromes are known to impair intellectual or cognitive performance, especially attention and concentration. In most studies, cognitive disturbances were not mentioned. The prevalence of cognitive complaints ranged from 20 to 68% (5, 12, 23–25). In a first comprehensive study (5), we demonstrated that all MMF patients had quantifiable neuropsychological involvement. MMF-associated cognitive dysfunction (MACD) is usually severe, and did not correlate to pain, fatigue, depression, disease duration, or drug intake (5, 6). Compared to control patients with arthritis and chronic pain, MMF patients had pronounced and specific cognitive impairment. The cognitive profile combines a dysexecutive syndrome, memory impairment, and signs of inter-hemispherical disconnection (5). Cognitive deficits did not correlate with pain, fatigue, depression, or disease duration. Most patients fulfilled criteria for non-amnestic/dysexecutive mild cognitive impairment (MCI) (Figure 2), even if some cognitive deficits seem unusually severe (6). MACD is probably the most disabling feature of MMF but seems stable with time, despite marked fluctuations (6). Long-term follow-up is needed to determine the evolution of patients.

#### Other manifestations

Psychiatric manifestations are unusually frequent in MMF patients, often leading physicians to consider physical complaints as psychosomatic. When checked on, the prevalence of mood disorders ranged from 50 to 60% (6, 25). MMF-associated psychiatric manifestations have not been specifically investigated, to date, but plausibly they could be consubstantial with the disease and contribute to alter the relations of patients with physicians. Various other complaints are reported by MMF patients, most regularly encountered being dyspnea and headache (12).

### Nature of the association between histological MMF lesions and symptoms

The genuineness of the association between general clinical manifestations and focal MMF histopathological lesion has been repeatedly disputed. However, since its first description, there are accumulating evidences in favor of a non-fortuitous association between the histological lesion and the symptoms presented by patients. First, in MMF patients, myalgias appear almost always subsequent to the administration of an aluminum hydroxide-containing vaccine (2). Second, the retrospective evaluation of 1292 consecutive patients who underwent deltoid muscle biopsy for diagnostic purposes showed a highly significant association (*p* < 0.0001) between chronic myalgias and the presence of MMF at biopsy (2). Interestingly, these findings go along the same lines of a previous large-scale survey identifying myalgias and arthralgias as adverse effects of anti-HBV vaccination (28). Third, a case–control study conducted on behalf of AFSSAPS (French equivalent of FDA) showed that (i) individuals with histological MMF lesions more frequently reported asthenia at onset than non-MMF individuals; and (ii) functional limitations due to fatigue were more important in MMF than in non-MMF individuals (19). Although only recently delineated, MACD is now recognized as a central feature in MMF, this dysfunction being much more frequent and severe than suspected by routine neurological evaluation (5). Importantly, it has been shown that non-specific factors such as pain, fatigue, depression, or drugs cannot explain by themselves the entire cognitive impairment and, compared to painful diseased controls, MMF patients displayed more severe cognitive deficits. The profile of neuropsychological dysfunction is comparable to that described in inflammatory or toxic conditions such as multiple sclerosis, human immunodeficiency virus (HIV) or hepatitis C virus (HCV) infections, or chronic aluminum exposure, supporting the view that MACD reflects an underlying organic, inflammatory, or toxic, brain involvement (5).

### Laboratory investigations in MMF patients

Positive diagnosis required to detect persistent MMF lesions at the surgical biopsy of deltoid muscle in adults and quadriceps muscle in children (see supra). Increased plasma creatine kinase (CK) levels may be observed in almost one third of patients. Unexceptionally, CK levels are increased at onset then get back to normal. Some patients have sustained increased CK levels; usually related to ongoing myonecrosis process with complement activation, usually ascribed to autoimmune mechanism. More generally, the presence of persisting elevated levels of CK should prompt the search of another cause of muscle involvement, especially inflammatory/dysimmune myopathy that may be associated with MMF (see infra). Baseline aluminum serum levels remain within reference values (2). However, through measurement of urinary aluminum excretion, it has been possible to demonstrate aluminum overload in one patient with vaccine-associated chronic fatigue syndrome and MMF (29). Electrodiagnostic testing may disclose myopathic feature in almost one-third of the patients. Skeletal muscle MRI is usually uninformative, except in case of associated diffuse myopathy.

In MMF patients, Gallium-67 (Ga^67^) scintigraphy disclosed a characteristic pattern of hyperfixations, mainly observed in fascias and periarticular areas in lower limbs, with a topography paralleling that of pain (21). These features are different from those observed in sarcoidosis (nodular lesions) and fibromyalgia (normal appearance). Gallium-67 (Ga^67^) is a radioisotope, which binds to the transferrin receptor (CD 71), which is especially expressed on the surface of macrophages and of different types of activated lymphocytes. However, the histopathological substratum of scintigraphic abnormalities in MMF has not been determined so far.

Because of cognitive disorders, most patients underwent routine brain MRI. Except in the subset of patients in whom MMF is associated with multiple sclerosis (9.3%) (12, 22), brain MRI appears poorly informative. Indeed, in a recent study, MRI was found normal/subnormal in 48% or showed non-specific brain supratentorial white matter T2-weighted hyperintensities in 38.5% (6). Other abnormalities included cortical (20%) or callosal (12%) atrophy. In contrast, functional imaging (single photon emission tomography, SPECT) was abnormal in 89%, hypoperfusions mainly affecting hippocampus, amygdala, and caudatus nucleus (6). When performed, evoked potentials (auditory, visual, and sensory) displayed abnormalities suggestive of central nervous system (CNS) demyelination in 38.5% MMF patients (6).

### Immunological abnormalities in MMF

Aluminum hydroxide is a potent activator of the immune system. In addition to their general symptoms, 19% of patients have an autoimmune disease characterized at time of MMF diagnosis, including multiple sclerosis, and also autoimmune thyroiditis, inclusion body myositis, dermatomyositis, rheumatoid arthritis, and Sjogren’s syndrome (12, 22, 30–32). Protracted immunological activation may be at the origin of arthralgias and the chronic fatigue syndrome (33) and the latter could be the result of augmented adjuvant effect of aluminum hydroxide-containing vaccines (34). These effects may be associated with a permanent production of proinflammatory cytokines [interleukin (IL)-1, IL-6, TNF-α, and GM-CSF], even if reported changes are excessively variable to be used for diagnostic purposes in the syndrome chronic fatigue (35–38). The MMF patients frequently have immunological abnormalities, in particular, an increase in the number of circulating B lymphocytes, and the presence of autoantibodies usually anti-nuclear and anti-phospholipid (unpublished data). Extensive cytokine screening showed increase of serum levels of the monocyte chemoattractant protein 1 (CCL2/MCP-1) in MMF patients compared to healthy subjects. MMF patients showed no elevation of other cytokines. This contrasted with inflammatory patients in whom CCL2/MCP-1 serum levels were unchanged, whereas several other inflammatory cytokines were elevated (39).

## Individual Susceptibility Factors

The existence of individual predisposing factors, not yet identified, to develop a long-lasting post-vaccination MMF associated with systemic symptoms is suggested by the small proportion of cases detected among vaccines (9). A genetic predisposition has been long suspected in patients, particularly on the basis of familial cases (23, 24, 40, 41). This view is supported by experimental results showing that genetic background is a key factor for the persistence of post-vaccinal granuloma (3). MMF is characterized by the increase of circulating CCL2/MCP-1, a cytokine implicated in the penetration of nanomaterials in brain (11, 39). The genotyping of 252 MMF patients and 516 controls in the CCL2 gene was in favor of the association between haplotype rs1024611/rs3760396 and the disease (Odds Ratio: 1,280, *p* = 0,088) (11). However, in 94 patients with the corresponding haplotype, the sequencing of 3 exons, 2 introns, and 3.5 kb upstream sequences in CCL2/MCP1 gene did not reveal anomalies. It seems plausible that CCL2 over expression results from a *trans* mechanism and pan-genomic approaches (G-WAS, exome) will be necessary to indentify the genetic variants associated with MMF.

## Autoimmune/Inflammatory Syndrome Associated with Adjuvants

In 2010, Shoenfeld and Agmon-Levin proposed the concept of *autoimmune/inflammatory syndrome induced adjuvants* (ASIA) that includes different clinical syndromes induced by exposure to xenobiotic having immune adjuvant properties (42). ASIA includes siliconosis (complications associated with silicone-containing implants), Gulf War syndrome, MMF, and post-vaccination phenomena. ASIA is characterized by a corpus of common symptoms including (i) muscle symptoms (myalgias, muscle weakness); (ii) joint involvement (arthralgia, arthritis); (iii) chronic fatigue and sleep disorders; and (iv) a neurological and/or cognitive involvement (Table 3).

**Table 3:** **Criteria for ASIA [from Shoenfeld and Agmon-Levin (42)]**.

Major criteria
Exposure to an external stimuli (Infection, vaccine, silicone, adjuvant) prior to clinical manifestations
The appearance of “typical” clinical manifestations
Myalgia, myositis, or muscle weakness
Arthralgia and/or arthritis
Chronic fatigue, un-refreshing sleep, or sleep disturbances
Neurological manifestations (especially associated with demyelination)
Cognitive impairment, memory loss
Pyrexia, dry mouth
Removal of inciting agent induces improvement
Typical biopsy of involved organs
Minor criteria
The appearance of autoantibodies or antibodies directed at the suspected adjuvant
Other clinical manifestations (i.e., irritable bowel syn.)
Specific HLA (i.e., HLA DRB1, HLA DQB1)
Evolvement of an autoimmune disease (i.e., MS, SSc)

## Conclusion

For decades, vaccines demonstrated their invaluable benefits in the fight against transmissible diseases. The repeated emergence of infectious threats crucially points out the need to have vaccines fully suitable for the widest possible population. The exact biological mechanisms leading to the outbreak of clinical manifestations in patients with long-term persisting MMF lesions are unknown so far. In the light of the story of MMF, one may consider that it is timely for health governmental agencies to rethink the methods for evaluating long-term safety of inorganic compounds and to support appropriate research programs.

## Conflict of Interest Statement

The authors declare that the research was conducted in the absence of any commercial or financial relationships that could be construed as a potential conflict of interest.
